# Technological solutions for reproducibility and “showing of work”: a response to “Redundancy of Terms Is Not an Error but Plays a Positive Role in Composing Search Strategies”

**DOI:** 10.5195/jmla.2020.936

**Published:** 2020-07-01

**Authors:** Tony Russell-Rose

**Affiliations:** 1 tgr2uk@gmail.com, Senior Lecturer in Computer Science, University of London; Director, UXLabs; and Founder, 2Dsearch, London, United Kingdom

## Abstract

Response to Schoones JW. Redundancy of terms is not an error but plays a positive role in composing search strategies [letter to the editor]. J Med Libr Assoc. 2020 Jan;108(1):118–9. DOI: 10.5195/jmla.2020.780. Comment on Salvador-Oliván JA, Marco-Cuenca G, Arquero-Avilés R. Errors in search strategies used in systematic reviews and their effects on information retrieval. J Med Libr Assoc. 2019 Apr;107(2):210–21. DOI: 10.5195/jmla.2019.567.

and

Salvador-Oliván JA, Marco-Cuenca G, Arquero-Avilés R. Response to “Redundancy of terms is not an error but plays a positive role in composing search strategies” [letter to the editor]. J Med Libr Assoc. 2020 Jan;108(1):118–9. DOI: 10.5195/jmla.2020.832.

**To the editor,** I read with interest the recent thread in the *Journal of the Medical Library Association* concerning the inclusion (or otherwise) of redundant terms in published search strategies [1, 2]. It seems that both correspondents agree on the value of “showing your work” in the development of search strategies but have differing views on the role of those intermediate solutions in the final published strategy. What is needed here is some mechanism to differentiate between the semantics of the final strategy and variations or alternative solutions that may be of interest to others wishing to learn from their work or otherwise extend it.

In my experience, this kind of requirement is, in general, poorly served by traditional databases and query formalisms. Moreover, I have long argued that the “Boolean string” as currently constituted has significant shortcomings regarding transparency, scalability, and robustness [3], and this contributes to many of the errors identified in the original article [4]. However, I believe we can learn much from solutions developed by related professions, in particular the computer science community who also need to create and manage complex logical artifacts in a transparent and reproducible manner. In software development, for example, this problem is solved through adopting formalisms that support comments and other annotations, so that additional variations can be included in the published version but play no part in the formal semantics.

In my own work, we are exploring ways to make structured searching more robust and reproducible and have developed formalisms that support this approach. You can see a simple example on using the 2Dsearch web application. This shows the Boolean string in question modeled as objects on a two-dimensional canvas. When represented in this form, a variety of novel transformations become possible, including the ability to “enable” and “disable” components on demand [3]. In this example, we see our original five terms, but the first four have been “disabled” (as indicated by the translucent rendering). As such they remain present and available for editing but play no part in the semantics of the executed query (which can be verified by opening the results pane). This approach can be applied to any search strategy or component, and the facility for naming individual search blocks further facilitates reusability and extension. It is my hope that solutions such as this, along with support for the sharing of strategies as executable objects in communal repositories, will eventually be adopted by the broader community and, thus, contribute to transparency and reproducibility in the evidence synthesis process.
